# Emerging technologies for engineering of extracellular vesicles

**DOI:** 10.3389/fbioe.2023.1298746

**Published:** 2023-11-09

**Authors:** Xin Zhou, Qing Huang, Yang Jiang, Huijing Tang, Luhan Zhang, Danyang Li, Yunsheng Xu

**Affiliations:** ^1^ Research Center, The Seventh Affiliated Hospital, Sun Yat-sen University, Shenzhen, China; ^2^ Department of Dermatovenereology, The Seventh Affiliated Hospital, Sun Yat-sen University, Shenzhen, China

**Keywords:** extracellular vesicle, engineering strategy, pre-treatment, parent cell, direct modification

## Abstract

Extracellular vesicles (EVs) are lipid-bilayer membrane-enclosed vesicles that are secreted by all cell types. Natural EVs contain biological information such as proteins, nucleic acids, and lipids from their parent cells. Therefore, EVs have been extensively studied as diagnostic biomarkers and therapeutic tools under normal and pathological conditions. However, some drawbacks, including low yield, poor therapeutic effects, lack of imaging, and targeting capacity of natural EVs, still need to be improved. Emerging engineering technologies have rendered EVs new properties or functionalities that broadened their applications in the biomedical field. Herein, in this review, we gave a brief overview of advanced strategies for EV engineering. We focused on pre-treatment of parent cells to regulate their released EVs. Meanwhile, we summarized and discussed the direct modification of EVs to achieve drug loading, imaging, and targeting functionalities for downstream applications.

## Introduction

Extracellular vesicles (EVs), including exosomes, microvesicles, and apoptotic bodies (Grange and Bussolati, 2022), are lipid-bilayer membrane-enclosed vesicles that are secreted by all cell types (Kalluri and LeBleu, 2020). EVs contain various biological molecules, such as proteins, nucleic acids, and lipids. These biological contents usually transfer from parent cells to recipient cells through receptor–ligand interaction, direct membrane fusion, and endocytosis or phagocytosis (Mathieu et al., 2019). In addition, the lipid-bilayer membrane of EVs not only serves as a protective barrier for the encapsulated bioactive molecules, ensuring their preservation and facilitating long-distance cellular communication, but also presents specific signals on the membrane surface that may help them skip macrophage-mediated clearance (Kamerkar et al., 2017). Therefore, EVs have been recognized as important mediator for cell-cell communications and potential therapeutic agents and diagnosis biomarkers (Liao et al., 2019).

Nevertheless, several challenges have been identified despite the prospective advantages of natural EVs. These include a limited yield from parent cells, weak therapeutic effects, and lack of imaging and targeting abilities. Therefore, researchers have shifted their focus toward engineered EVs.

**FIGURE 1:**
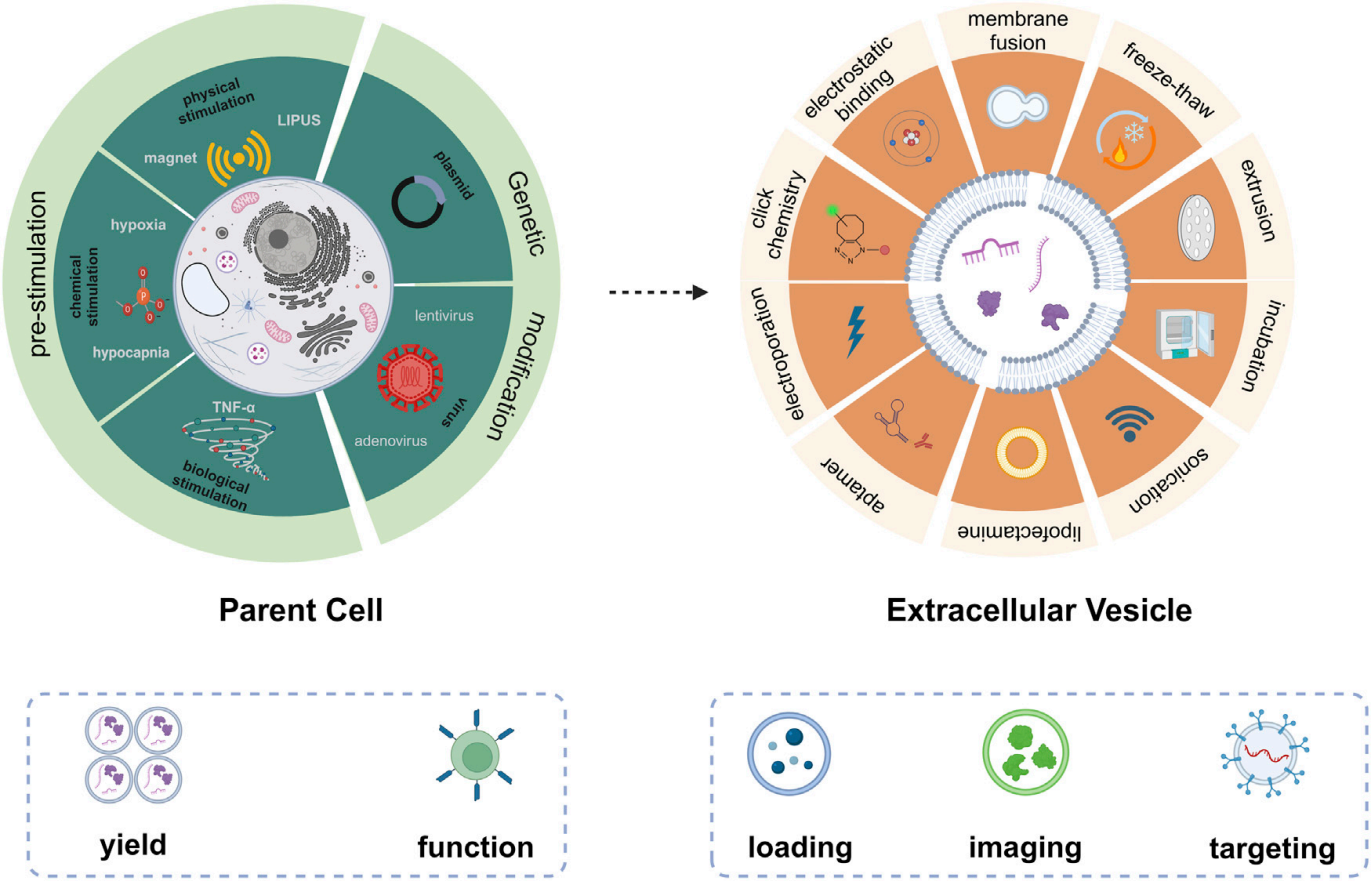
Emerging technologies for engineering of EVs based on pre-treatment of parent cells (left) and direct modification of EVs (right).

Herein, we summarize the emerging approaches for engineering EVs (Figure 1). These include pre-treatment of parent cells and direct modification of EVs. Pre-treatment of parent cells, e.g., gene edition and pre-stimulation of parent cells, renders EVs with more attractive bio-functions and higher yield. Direct engineering of EVs amplified the downstream applications, such as drug loading, imaging, and targeting. We hope this minireview can provide some valuable hints and references for the engineering strategies of natural EVs to achieve smarter EVs for biomedical applications.

## Engineering technologies based on pre-treatment of parent cells

According to the biogenesis of EVs, the specificity and abundance of EV cargoes were intrinsically linked to the cell type and gene expression of parent cells. Typically, genetic engineering and pre-stimulation of parent cells (Table 1) were employed to modulate their gene expression. This has led to the alteration of nucleic acids, proteins, and lipids, which were subsequently packaged into EVs, endowing EVs with various biomedical functions.

**TABLE 1:** A summary of commonly used approaches (pre-stimulation and genetic modification of parent cells) to produce engineered EVs.

Type	Approach	Parent cell	Function of EVs	Mechanism	References
Biological stimulation	TNF-α	Human gingival MSCs	Anti-apoptosis of retinal ganglion cells *in vivo* and *in vitro*. Anti-inflammation of LPS-activated BV2 *in vitro*	MEG3/miR-21-5p/PDCD4 Pathway	Yu et al. (2022)
	TNF-α	Human gingival MSCs	Reducing bone loss in periodontitis model *in vivo*. Anti-inflammation via switch M1 into M2 *in vitro*	Wint5a and RANKL/OPG	Nakao et al. (2021)
	Insulin-like growth factor-1	Neural stem cell	Inhibiting neural apoptosis in PC12 cells *in vitro*. Pro-proliferation of neural *in vivo*	miR-219a-2-3p/YY1	Ma et al. (2019)
	Meurotrophic factor	Human umbilical cord MSCs	Promoting the recovery of cognitive and sensorimotor function *in vivo*. Increasing the number of neuN+, MBP+ cells *in vivo*	N/A	Liu et al. (2023)
Chemical stimulation	Hypoxia	Dental pulp stem cells	Anti-inflammation via switch M1 into M2 *in vitro* and vivo. Inhibiting osteoclast formation *in vitro* and *in vivo*	MiR-210-3p targeting NF-κβ1 and p105	Tian et al. (2023)
	Hypoxia	MSCs	Promoting cell proliferation, migration, and tube formation of HUVEC *in vitro*. Pro-angiogenesis in the injured spinal cord *in vivo*	HIF-1α and VEGF	Mu et al. (2022)
	Hypocapnia	Adipose-derived stem cells	Enhancing the migration of PC 12 cells *in vitro*. Improving nerve regeneration *in vivo*	MiR-218	Wang et al. (2022)
Physical stimulation	LIPUS	Schwann cells	Enhancing the axon elongation. Promoting the injured CN regeneration and Schwann cell proliferation	PI3K/Akt/Fox signaling pathway	Ye et al. (2023)
	Magnet	Schwann cells	Switching the on/off of the release of EVs	N/A	Xia et al. (2023)
	Mechanical force	MSCs	Increasing the yield of EVs by up to 4 - 5.7 fold	N/A	Yan et al. (2021)
Genetic modification	XStamp-PDGFA lentiviral vector	Neural stem cells	Target ability by PDGF-A. Increasing the numbers of OLGs and blockading	N/A	Xiao et al. (2022)
	GFP-CTF1 adenoviral vector	Bone MSCs	Demyelination *in vivo*. Promoting myelination *in vivo*	JAK/PI3K/mTOR/STAT3 signaling pathway	Zhu et al. (2022)
	CD63 Plasmid	HEK-293T cells	Promoting migration, proliferation, and angiogenesis of HUVEC *in vitro*	N/A	Kojima et al. (2018)

### Pre-stimulation of parent cells for EVs engineering

Cell functions and phenotypes were influenced by various factors in the conditional culture micro-environment, including biological, chemical, or physical stimulation. Therefore, pre-stimulation of parent cells has been extensively employed as an advanced approach for EV engineering.

Bioactive factors, such as cytokines, growth factors, and chemokines, can rapidly influence cellular activity via ligand-receptor interactions. Culturing parent cells with pro-inflammatory cytokine, e.g., TNF-α, was often employed as an EV engineering strategy. For instance, mesenchymal stem cells (MSCs) treated with TNF-α demonstrated an enhanced ability for immune response to challenging microenvironments. Yu et al. (2022) showed that engineered EVs derived from gingival MSCs treated with 10 ng/ml of TNF-α presented the neuroprotective effects through the MEG3/miR-21a-5p axis. These engineered EVs significantly alleviated the survival of retinal ganglion cells and inhibited inflammation in BV2 cells. Similarly, Nakao et al. (2021) found that EVs derived from preconditioning gingival MSCs with 100 ng/ml of TNF-α effectively mitigated bone loss in a ligature-induced periodontitis model. However, several studies noted that EVs can mimic the same biomedical effects as the added stimulation in their parent cell cultures. For example, neurotrophic factor has a neuroprotective effect. After adding 30 ng/ml of neurotrophic factor to the culture medium for 48 h, EVs derived from human umbilical cord MSCs fostered cognitive and sensorimotor function recovery in a mouse model with traumatic brain injury. Also, the count of NeuN+ (labeled neurons) and MBP+ (labeled myelin sheath) cells was increased in the engineered EVs group (Liu et al., 2023). Pre-stimulation of parent cells could also change the content of EVs. Compared to natural EVs, miR-219a-2-3p levels were elevated in insulin-like growth factor-1 treated EVs. Following this, miR-219a-3p targeted both Yin Yang 1 (YY1) and p65, leading to the proliferation of neural cells in the spinal cord injury model (Ma et al., 2019).

Hypoxic stimulation was a widely used strategy to amplify the therapeutic effects of EVs. Briefly, once normal cultured cells achieve 80%–90% confluence, they were then cultured in a serum-free medium for 48 h under 1% O_2_ conditions to induce hypoxia. MiRNA profiles from dental pulp stem cell-derived EVs indicate that miR-210-3p was more abundant in hypoxic EVs than natural EVs. MiR-210-3p targets the NF-κβ signaling pathway in macrophages both *in vitro* and *in vivo*, thereby mitigating inflammatory osteolysis (J. Tian et al., 2023). Furthermore, EVs derived from MSCs promoted angiogenesis when the parent cells undergo hypoxic preconditioning by transferring HIF-1α to vascular endothelial cells (Mu et al., 2022). Hypocapnia represents another form of chemical stimulation that expands the application scope of EVs in regenerative medicine. For instance, Wang et al. reported that EVs derived from hypocapnia (3% CO_2_ condition) stimulation adipose stem cells enhance nerve regeneration in a mouse model with spinal cord injury. This process is potentially mediated by controlling nerve cell migration and the delivery of miR-218 from hypocapnia EVs (Wang et al., 2022).

Apart from their therapeutic effects, the low yield of EVs is a significant limitation to the applications of EV-based therapeutics. Emerging physical stimulation approaches have enabled parent cells to produce a higher yield of EVs and even turn their secretion on/off. Yan et al. (2021) discovered a novel EV bioreactor where the mechanical force generated from a rotary cell culture system at 36 rpm/min amplified EV production up to 4-fold. Similarly, de Almeida Fuzeta et al. (2020) utilized a vertical-wheel bioreactor to culture MSCs and observed that shear stress increased both surface tensile strength and membrane contraction, which resulted in a 5.7-fold increase in EV productivity. Other researchers also introduced a magnetic-responsive platform termed Mag-gel. Depending on the presence or absence of micro/nano-scale force from a rotating magnetic field, Schwann cells can effectively switch the secretion process of EVs on or off (Xia et al., 2023).

### Genetic modification of parent cells for EV engineering

Genetic modification stands as a cornerstone technique in various biomedical domains, allowing for precise manipulation of gene expression of cells. It was evident that numerous researchers have endeavored to introduce both native and non-native functional oligonucleotides into parent cells to modulate gene expression. Consequently, cellular products from gene editing cells, such as DNA, RNA, lipids, and proteins, might be encapsulated within the membrane or the lumen of EVs.

For instance, Xiao et al. (2022) constructed EVs targeting the central nervous system (CNS) by introducing the fragment of platelet-derived growth factor A (PDGFA) into neural stem cells via lentivirus. Compared with unmodified EVs, these engineered EVs displayed increased affinity towards primary oligodendrocyte progenitor cells *in vitro*. Meanwhile, intense fluorescence signals were observed in the spinal cord and brain *in vivo*. Thus, PDGFA modification by lentiviral transfection enhanced the targeting capability of EVs. In another study, cardiotrophin-1 (CTF1) was overexpressed in bone marrow-derived MSCs via transfection with adenoviral vector (Zhu et al., 2022). The subsequently obtained EVs were also characterized with highly expressed CTF1. In another study, hFOB1.19 cells were transfected with maternally expressed gene 3 (MEG3) via Lipofectamine 3000 for the upregulation of lncRNA MEG3 in both parent cells and corresponding EVs (Huang et al., 2022). In clinical osteosarcoma samples, lncRNA MEG3 displayed diminished expression compared to non-tumorous tissues. Therefore, they delivered EVs containing MEG3 to an osteosarcoma mouse model, resulting in pronounced anti-tumor effects, evidenced by reduced proliferation, migration, and increased apoptosis of tumor cells.

Nonetheless, the limited efficiency with which EVs package functional molecules from their parent cells remains challenging. Some investigations have enhanced this efficiency by editing genes associated with the structural protein of EVs. Tetraspanins were characterized to have four transmembrane domains, three intravesicle segments, and two extravesicle loops. In EVs, tetraspanins such as CD9, CD63, and CD81 were prime candidates for genetic modification. For example, Kojima et al. (2018) attached therapeutic mRNA to the C-terminutesus of CD63, which subsequently alleviated neurotoxicity in Parkinson’s disease models and LPS-induced microglial cells. A pH-responsive CD63-pHluorin-engineered EV was also introduced. When EVs transitioned from the multivesicular bodies’ acidic environment (pH = 5.5) to the neutral extracellular matrix (pH = 7.4), the vesicles immediately emitted green fluorescence (Verweij et al., 2018). The combination of target sequences and structural genes of EVs presents a pioneering strategy for consistently generating engineered EVs.

## Emerging engineering technologies based on direct modification of EVs

Numerous bioactive molecules can be synthesized *de novo* at cellular level. Following, those bioactive molecules were possibly packaged into EVs. However, synthesizing small molecule drugs, fluorescent probes, and target peptides in parent cells was challenging. Hence, apart from focusing on parent cells, direct modification of EVs offers another avenue for addressing desired usages such as drug loading, imaging, and targeting.

### Engineering EVs for drug loading

EVs were notable for their low immunogenicity, high biocompatibility, circulation stability, and ability to cross physiological barriers effectively. Recently, their potential as drug carriers has gained significant attentions. A key challenge was to bypass the EV membrane for efficient drug loading. Techniques to encapsulate small molecule drugs into EVs included incubation, electroporation, extrusion, sonication, freeze-thaw cycles, and lipofection.

**FIGURE 2:**
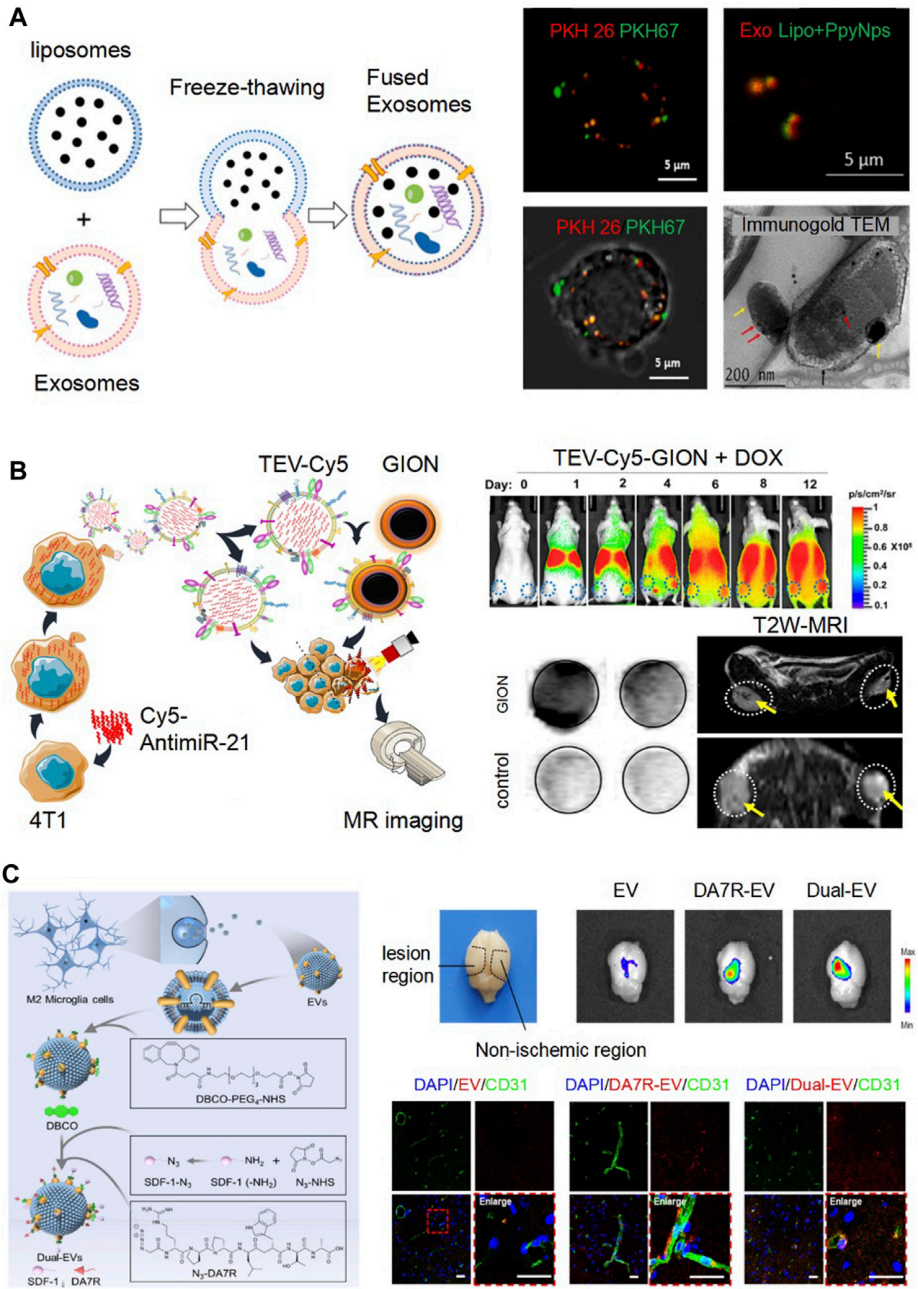
Emerging engineering technologies based on direct modification of EVs. **(A)**. Fusing EVs and liposomes by freeze and thawing for drug loading. Confocal fluorescence images exhibited a fuse of EVs stained with PKH-26 dye (red) and gold nanoparticle-laden liposomes (Lipo + PpyNps) labeled with PKH-67 dye (green). Immunogold TEM showed that EVs (red arrow) and Lipo + PpyNps (yellow arrow) were fused together. Reproduced with permission (Singh et al., 2021). Copyright 2021 Elsevier. **(B)**. TEV-Cy5 were loaded GION as imaging probes by membrane fusion (TEV-Cy5-GION). From day 2–12, TEV-Cy5-GION steadily accumulated in the tumor by fluorescence imaging. By the 12th day, the MRI signal change in the tumor region displayed a pronounced T2 signal reduction in mice treated with TEV-Cy5-GION, in contrast to the control group. Reproduced with permission (Jc Bose et al., 2018). Copyright 2018 American Chemical Society. **(C)**. Binding DA7R and SDF-1 to Dual-EVs using click chemistry for targeting delivery. The fluorescence of the engineered EVs labeled with Cy5.5 was predominantly detected in the lesion area. By immunofluorescence staining, the engineered EVs predominately accumulated at the ischemic site, co-localizing with CD31-marked blood vessels. Reproduced with permission (Ruan et al., 2023). Copyright 2023 Elsevier.

Gong et al. (2019) incubated EVs with Doxorubicin (DOX) overnight and observed variable loading efficiencies across different Dox concentrations (100∼1,000 μg/ml). The maximal loading efficiency was achieved at a concentration of 200 μg/ml. Subsequently, they introduced hydrophobic miR-159, modified it with cholesterol, and incubated it for 90 min. Through fluorescent quantification, they recorded an approximately 4.5-fold increase in loading efficiency. However, this incubation method was often considered a passive strategy and tended to offer lower encapsulation efficiencies. To enhance drug packaging efficiency, sonication and incubation were compared (Gao et al., 2021). Notably, after sonicating a mixture of EVs and berberine 20 times, there was a 3.5-fold increase in loading efficiency. Analysis revealed no significant changes in the zeta potential or size between the berberine-loaded EVs (EVs@Ber). A 7-day stability test also confirmed no remarkable change in the size and zeta potential of the EVs@Ber, indicating their stability. Incorporating water-soluble drugs into EVs remains challenging. Electroporation was introduced for the loading of water-soluble drugs, polyoxotungstate (POM1) and metformin (Met), which were positively charged, into cancer cell-derived EVs (C-EVs) (Wu et al., 2022). By analyzing the zeta potential of both the untreated C-EVs and drug-loaded C-EVs (C-EV@PMet), they discovered that C-EV@PMet had a higher zeta potential, indicating successful packaging of POM1 and Met into the C-EVs. Furthermore, Met, a crucial enzyme in ATP synthesis, has been shown to enhance the accumulation of pro-inflammatory ATP and decrease the levels of immunosuppressive adenosine, thereby influencing the tumor’s metabolic microenvironment. In a separate study, Singh et al. (2021) employed a freeze-thaw method to engineer EVs with liposomes loaded with drugs. Specifically, liposomes were combined with EVs in a 1:2 ratio and then subjected to ten freeze-thaw cycles using liquid nitrogen and a 50°C water bath. Confocal fluorescence images exhibited a fuse of EVs stained with PKH-26 dye (red) and gold nanoparticle-laden liposomes labeled with PKH-67 dye (green), which was also corroborated by immunogold TEM (Figure 2A). Fan et al. (2023) presented an efficient engineering strategy for milk-derived EVs using extrusion. In their method, EVs and drugs were mixed at a ratio of 2:1 and then successively extruded through polycarbonate films with sizes of 1 μm, 400 nm, and 200 nm. As a result, they achieved engineered EVs with a final size of 255 nm. Briefly, considering the varied physicochemical properties of drugs and the following application, the appropriate EV engineering approaches should be chosen to maximize drug-loading efficiency and maintain the bio-function of EVs and drugs.

### Engineering EVs for imaging

The tracking, distribution, and pharmacokinetic profiles of EVs post-administration *in vivo* remain elusive, posing challenges to developing precision therapy. Given EVs’ positive and passive accumulation capabilities to disease tissues or organisms, leveraging engineered EVs for imaging has emerged as a novel strategy. In this context, we provided a succinct overview of various imaging modalities and their associated engineering approaches based on EVs, including fluorescence imaging, radionuclide imaging, and magnetic resonance imaging.

To observe EVs distribution, EVs were treated with the lipophilic dye DiD. Fluorescence imaging analysis of the dissected organs revealed a positive signal within the tumor after 24 h of post-administration (Garofalo et al., 2019). However, fluorescence imaging faces challenges, such as auto-fluorescence and limited tissue penetration depth during live imaging. The advanced techniques of radionuclide imaging and magnetic resonance imaging offer better resolution and sensitivity. Faruqu et al. (2019) developed two methods to label ^111^In to B16F10-derived EVs for assessing tumor accumulation of EVs. A comparison of tissue biodistribution at 24 h showed a notable difference between [^111^In] tropolone-EVs of intraluminal radiolabeling and [^111^In]DTPA-EVs of membrane radiolabeling. The latter displayed higher accumulation in the liver, spleen, and tumor. In conclusion, membrane radiolabeling demonstrated superior efficiency and stability compared to intraluminal radiolabeling. In Figure 2B, EVs were engineered to combine Cy5 fluorescence and gold−iron oxide nanoparticles (GION) as imaging probes (TEV-Cy5-GION), which exhibited remarkable fluorescence and MRI T2 contrast. From day 2–12, TEV-Cy5-GION were accumulated in the tumor. The MRI signal change in the tumor region displayed a pronounced T2 signal reduction in mice treated with TEV-Cy5-GION on day 12, in contrast to the control group (Jc Bose et al., 2018).

### Engineering EVs for targeting

Targeted delivery stands as a crucial component in precision medicine. Nevertheless, unmodified EVs often demonstrate inefficiencies in targeted delivery, frequently cleared by macrophages or internalized by non-target tissue cells. Consequently, the engineered EVs become imperative to enhance targeted delivery. In this section, we discussed the principal engineering strategies to render EVs with targeting ability. These strategies can be divided into physical or chemical methods, consisting of membrane fusion, electrostatic binding, click chemistry, etc.

Physical modifications employed physical force to disrupt the lipid membrane structure temporarily. For instance, Li et al. (2022) designed engineered EVs using a fusion approach. They synthesized liposomes through assembly with phospholipid polyethylene glycol RGD (DSPE-PEG-cRGD). Subsequently, the liposomes and EVs were extruded through membrane fusion. The incorporated RGD of engineered EVs offered tumor targeting capability, as it bound with integrin receptors on tumor surfaces. Typically, EVs possess a negative charge, allowing them to bind with positively charged particles via electrostatic binding. A straightforward and efficient method was reported by modifying EVs through incubation. As the cationized pullulan concentration increased, the engineered EVs’ zeta potential progressively shifted from negative to positive (Tamura et al., 2017).

Click chemistry, a notable technology, has been extensively employed for the surface modification of engineered EVs. Ruan et al. (2023) showcased engineered EVs targeting the ischemic regions in stroke mice (Figure 2C). Employing copper-free click chemistry, they modified the EVs with both the high-affinity ligand for damaged blood vessels, DA7R (^D^R^D^P^D^P^D^L^D^W^D^T^D^A) and the stromal cell-derived factor-1a (SDF-1). As a result, the fluorescence of the engineered EVs labeled with Cy5.5 was predominantly detected in the lesion area, as opposed to the non-ischemic region in the stroke model mice. In addition, as illustrated in Figure 2C, the engineered EVs predominantly accumulated at the ischemic site, with 58% co-localizing with CD31-marked blood vessels, which is a significant increase when compared to the mere ∼14.9% observed with unmodified EVs.

Herein, direct modification of EVs with targeting molecules represents an effective strategy for targeted delivery. The selection of the optimal method to bind the target molecule to the EVs should be based on the specific properties of the target molecule.

## Conclusion and perspective

Natural EVs were abundant in diverse biomolecules contained on the membrane surface within their lumen. Assisted with advanced technologies, engineered EVs have exhibited broader applications and potentials in biomedical field. In this minireview, we briefly summarized the emerging approaches for EVs engineering including pre-treatments of parent cells and direct modification of EVs. Pre-treatments of parent cells by biological, chemical, and physical stimulation and genetic modification could potentially enhance the production yield and therapeutic potency of EVs. We have also summarized the advanced strategies for direct modifications of EVs for downstream applications, such as for drug loading, as imaging tools, and enhancing targeting ability.

Despite the extensive research focused on engineering approaches for EVs over the years, certain challenges still existed. Firstly, the loading efficiency for drug or imaging agents was unsatisfactory. This limitation may lead to the requirement for administration of higher amount of engineered EVs to achieve therapeutic or imaging effects, thereby posing a potential bio-safety risk due to excess EVs. Thus, seeking better approaches to improve the loading efficient is in urgent need. Secondly, to keep the bio-function of EVs, preservation EVs’ membrane integrity during the engineering process should be considered. Therefore, developing milder engineering conditions is also important for their biological applications. Lastly, there was no doubt that numerous engineering strategies had enhanced the therapeutic efficacy of EVs, but biosafety and mechanisms of engineered EVs remained unclear. Hence, more studies should be employed to evaluate the pros and cons of engineering strategies for EVs.

Overall, advanced engineering strategies have endowed EVs more possibilities for clinical translation. With continued research and innovation, we are optimistic that engineering EVs will pave the way for breakthroughs in precision medicine.
